# Limitations and possibilities of low cell number ChIP-seq

**DOI:** 10.1186/1471-2164-13-645

**Published:** 2012-11-21

**Authors:** Gregor D Gilfillan, Timothy Hughes, Ying Sheng, Hanne S Hjorthaug, Tobias Straub, Kristina Gervin, Jennifer R Harris, Dag E Undlien, Robert Lyle

**Affiliations:** 1 Department of Medical Genetics, Oslo University Hospital, Oslo, Norway; 2 Ludwig Maximilians Universität, Adolf Butenandt Institut, Lehrstuhl für Molekularbiologie, Schillerstraße 44, München, 80336, Germany; 3 Department of Medical Genetics, Oslo University Hospital and University of Oslo, Oslo, Norway; 4 Division of Epidemiology, Norwegian Institute of Public Health, Oslo, Norway

**Keywords:** PCR duplicates, Redundant reads, HTS, NGS, Next generation sequencing, Micro-ChIP, N-ChIP, Native ChIP, Location analysis, Histone

## Abstract

**Background:**

Chromatin immunoprecipitation coupled with high-throughput DNA sequencing (ChIP-seq) offers high resolution, genome-wide analysis of DNA-protein interactions. However, current standard methods require abundant starting material in the range of 1–20 million cells per immunoprecipitation, and remain a bottleneck to the acquisition of biologically relevant epigenetic data. Using a ChIP-seq protocol optimised for low cell numbers (down to 100,000 cells / IP), we examined the performance of the ChIP-seq technique on a series of decreasing cell numbers.

**Results:**

We present an enhanced native ChIP-seq method tailored to low cell numbers that represents a 200-fold reduction in input requirements over existing protocols. The protocol was tested over a range of starting cell numbers covering three orders of magnitude, enabling determination of the lower limit of the technique. At low input cell numbers, increased levels of unmapped and duplicate reads reduce the number of unique reads generated, and can drive up sequencing costs and affect sensitivity if ChIP is attempted from too few cells.

**Conclusions:**

The optimised method presented here considerably reduces the input requirements for performing native ChIP-seq. It extends the applicability of the technique to isolated primary cells and rare cell populations (e.g. biobank samples, stem cells), and in many cases will alleviate the need for cell culture and any associated alteration of epigenetic marks. However, this study highlights a challenge inherent to ChIP-seq from low cell numbers: as cell input numbers fall, levels of unmapped sequence reads and PCR-generated duplicate reads rise. We discuss a number of solutions to overcome the effects of reducing cell number that may aid further improvements to ChIP performance.

## Background

Chromatin immunoprecipitation (ChIP) is used to determine the genomic interaction sites between nuclear proteins and nucleic acids. Standard ChIP protocols used for genome-wide studies typically require large quantities of starting material, in the range of 10^7^ cells. The amount of material immunoprecipitated varies depending on the target protein and antibody employed, but is usually in the range of a few hundred picograms to tens of nanograms.

Over the past decade, genome-wide analysis of ChIP material has been possible by employing whole genome amplification techniques to produce microgram quantities of DNA for hybridisation to microarrays, commonly referred to as ChIP-chip. Recently, the application of high-throughput sequencing (HTS) to analyse the immunoprecipitates, commonly referred to as ChIP-seq, has replaced ChIP-chip as the preferred means of harvesting genome-wide data, and confers a number of advantages including higher resolution, improved quantification range, greater genome coverage, fewer artifacts, and lower cost [1]. However, the library preparation methods required to render immunoprecipitated DNA ready for HTS sequencing involve inefficient enzymatic steps and multiple purifications, each resulting in sample loss. As a result, ChIP-seq requires a similar starting amount of immunoprecipitated DNA (1–10 ng) to ChIP-chip, and nonetheless involves many cycles of PCR (typically 15–18 cycles). These limitations mean that it is still challenging to apply ChIP-seq to low numbers of cells.

There have been several recent papers detailing ChIP protocols applicable to low cell numbers (down to 100 cells) based on the inclusion of carrier DNA [2] or improvements in the efficiency of the immunoprecipitations themselves [3-7]. However, these techniques have been limited to locus-specific analysis of the precipitates by quantitative PCR. By incorporating whole genome amplification (WGA) methods, these techniques have been extended to allow genome-wide analysis by ChIP-chip from as few as 10,000 cells [8] or 1,000 cells [9].

Three recent publications detail the development of ChIP-seq protocols for use with low cell numbers, all based on formaldehyde crosslinked chromatin: Using standard Illumina library preparation procedures, Hitchler & Rice demonstrated ChIP-seq from 1 × 10^6^ human stem cells and 5 ng immunoprecipitated DNA [10]. Greater reductions have been possible using alternative library preparation methods, where cell requirements have been lowered to 10,000 [11] (the method is also presented in more detail elsewhere [12]), and even 5000 [13], with input DNA amounts below 50 pg. However, these methods employ either lengthy linear amplification procedures, or primer extension (4 cycles) and 15 cycles of PCR – all prior to a standard Illumina library prep entailing a further 17–18 cycles of PCR.

An alternative method of performing ChIP avoids the use of formaldehyde cross-linking and is thus known as native ChIP (N-ChIP) [14-17]. The advantages of N-ChIP over cross-linked ChIP (X-ChIP) include higher resolution, and lack of unspecific interaction caused by formaldehyde crosslinking. It has also been suggested that the N-ChIP method is more sensitive than X-ChIP, as epitopes may not be masked by cross-linked proteins or be themselves denatured by formaldehyde [15] making N-ChIP ideally suited to studies aiming to examine small cell numbers. However, N-ChIP is generally considered only applicable to histone proteins, although successful N-ChIP of MeCP2 [18] and a handful of transcription factors including Runx2, Dlx6 and Sin3a have been reported [19].

We present here an N-ChIP method for genome-wide analysis by ChIP-seq, optimised for use with low starting cell numbers (here 200,000, divided into two immunoprecipitations of 100,000 each). This demonstrates ChIP-seq with 200-times fewer cells than a previously published method used as a benchmark for comparison. The performance of the optimised method was evaluated for read mapping, sensitivity and specificity at a range of starting cell numbers covering three orders of magnitude, starting with the published amount of 2 × 10^7^ cells / IP and reduced to a point where sensitivity was compromised, to determine the limits of the technique.

## Results

The ChIP method described here was developed using the N-ChIP method of Zhao and colleagues [20,21] as a starting point. We therefore set the existing technique, using the published amount of 2 × 10^7^ cells per immunoprecipitation, as a benchmark against which to compare the performance of our method at a series of decreasing cell numbers. The new method presented here significantly shortens the procedure by eliminating the need for dialysis, and incorporates modifications optimised for low cell numbers.

Chromatin prepared using the two methods was prepared from cultured CD4^+^ lymphocytes, and immunoprecipitated with anti-H3K4me3 antibody. Enrichment at positive and negative control loci (see methods section) was measured by quantitative PCR prior to generation of Illumina sequencing libraries. Each ChIP-seq library was then sequenced on a single lane of an Illumina GAIIx sequencer, and generated chromatin profiles typical of H3K4 trimethylation, from which peaks were called. At this point it was determined that additional sequencing was required to saturate peak calling (see below) in the lowest cell number employed, so additional sequencing was performed for this sample using a single lane of an Illumina HiSeq 2000 machine.

**Figure 1:**
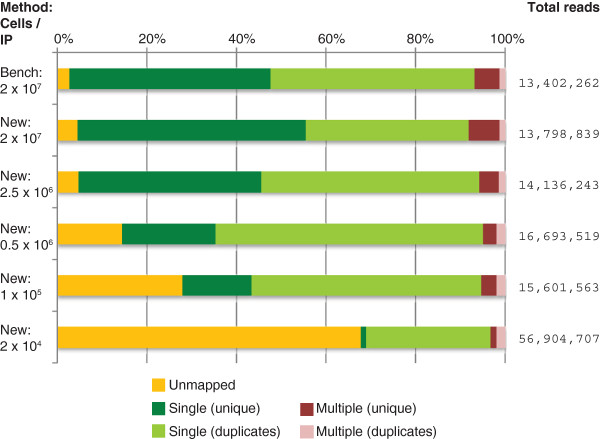
**Genomic mapping of sequence reads.** The proportion of reads that were unmapped, those mapping to single genomic positions, and those mapping to multiple locations (repeats) are illustrated. The latter two categories are broken down into reads present as a unique copy, or those reads that are present in two or more identical copies (duplicates). The total number of reads generated for each experimental condition are given at the right.

The total number of reads generated for each library and the results of aligning these libraries to the human genome are summarized in Figure 1. As cell numbers are reduced, the number of unmapped reads increases. In addition, the percentage of mapped reads derived from duplicate reads increases. A sample of unmapped reads was aligned against the GenBank nucleotide database [22] using blast [23]. The results showed that a small proportion of unmapped reads in all cases represent those containing sequencing errors that fail to map to the human genome (the BWA algorithm does not tolerate more than 2 mismatches in its 32 bp seed). The remainder of unmapped reads, which increase with decreasing input cell number, fail to map with high confidence to any sequence in the GenBank database and are apparently PCR amplification artifacts. The increased level of duplicate reads seen at lower cell numbers is also assumed to be introduced during the PCR amplification (18 cycles) required as part of Illumina library preparation. The same number of PCR cycles were applied to each sample, to facilitate inter-sample comparisons. A disadvantage of this approach is that a greater number of cycles than necessary to generate sufficient library to sequence, were applied to the higher cell number samples. Despite this, only in the low cell number samples did the decreased amount and complexity of the input material, lead to high proportions of duplication during amplification.

**Figure 2:**
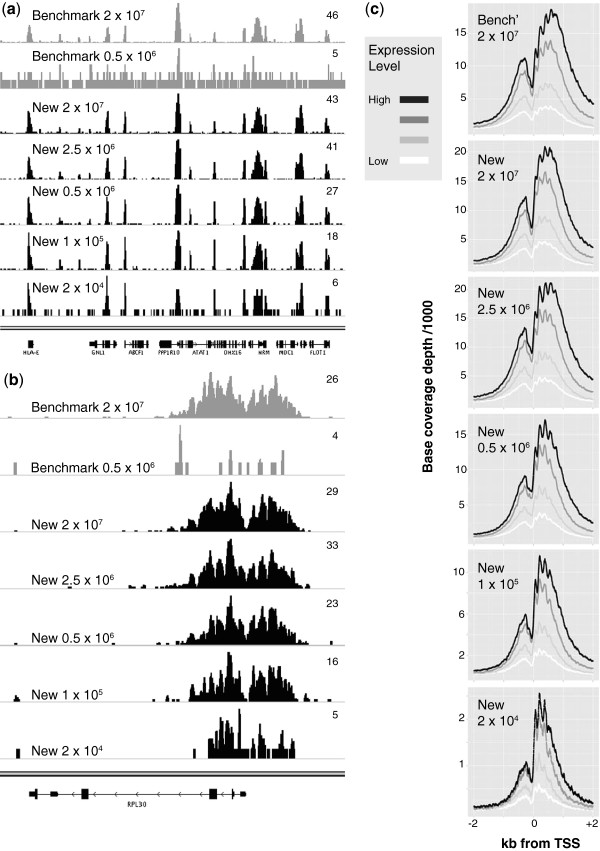
**H3K4me3 peaks are found at promoters, where peak heights parallel gene expression levels.** (**a**) 330 kb section of the gene-dense major histocompatibility complex (MHC) visualised in the Integrative Genomics Viewer [25]. Tracks display read depth for benchmark (gray) and new (black) ChIP methods at decreasing input cell numbers. Maximum read depth over the displayed area is indicated on the right of each track. Only uniquely mapping, non-duplicate reads are displayed. (**b**) 8 kb region showing H3K4me3 signal over the promoter of the *RPL30* gene. (**c**) Sequence coverage over transcription start sites (TSS). Coverage is displayed as a function of gene expression, with genes divided into quartiles based on expression level.

ChIP-seq profiles from each experiment can be seen in Figure 2a and 2b, and by visual inspection, peaks of H3K4me3 can be seen to occupy promoter regions as expected from previous studies [24]. To demonstrate the increased sensitivity of the new method relative to the benchmark, an additional profile generated using the benchmark at a low cell number is shown (Figure 2a and 2b). The resolution afforded by the MNase digestion allows identification of individual nucleosome positions (Figure 2b). The relationship between H3K4me3 and transcription start sites (TSS) was confirmed by plotting H3K4me3 levels relative to transcription start sites (Figure 2c), which reproduces earlier findings showing depletion of histones at the TSS itself, and a series of clearly positioned nucleosomes upstream and downstream [20]. Furthermore, immunoprecipitation with H3K4me3 is strongest at highly expressed genes, and diminishes with decreasing expression level (Figure 2c).

**Figure 3:**
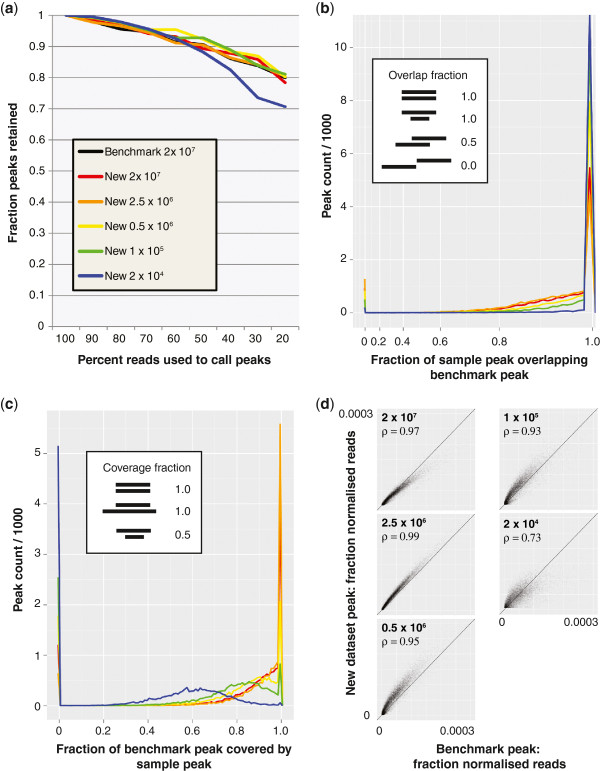
**Saturation, sensitivity and correlation of peak calling with decreasing cell number.** (**a**) Saturation of peak calling as reads are randomly discarded. Peaks were called using only unique non-duplicated reads. (**b**) Overlap of called peaks in the different datasets with benchmark dataset peaks. Inset diagram defines examples of full or partial peak overlap, with the upper bar in each case representing the benchmark. Colours as in panel a. (**c**) Coverage of benchmark peaks by peaks in other datasets. Colours as in panel a. Inset shows examples of coverage, with upper bar in each case representing the benchmark. (**d**) Correlation of peak heights between benchmark and new sample datasets. Spearman correlation coefficients (ρ) are given. Only peaks overlapping a benchmark peak were included in this analysis. The number of reads in a given peak was normalised to the total number of reads (uniquely mapping non-duplicated) in the sample.

To define genomic regions of H3K4 trimethylation, peak calling was performed using MACS [26], using only uniquely mapping, non-duplicate reads. Performing peak calling whilst including duplicate reads led to the appearance of high numbers of non-specific peaks, particularly in the lowest cell number sample (data not shown). Calling peaks when correcting for background control (sequencing libraries prepared from each sample’s ChIP input DNA) made negligible difference to the number of peaks recognised; 0.03 – 1.43% of peaks were no longer called when using a control dataset. The total number of peaks called for each cell number is summarized in Table 1. The number of peaks called falls appreciably only at the lowest cell input number tested, to below 75% of the number called in the benchmark. Crucially, despite the lower numbers of uniquely mapped reads recovered from the lowest cell number sample, peak calling was saturated for all samples: When decreasing proportions of total available reads were used to call peaks (Figure 3a), the absence of a reciprocal relationship between read count and peaks called indicates that all samples are approaching saturation (i.e. where adding more reads will not increase the number of peaks called). Therefore, the lower number of peaks called using only 20,000 cells / IP is due to the reduced number of useful reads (non duplicated and uniquely mapping) available as cell numbers fall, and not simply because more sequencing is required.

**Table 1:** Peak calling, sensitivity (detection of peaks called in the benchmark) and specificity (off-target peaks not present in the benchmark)

**Protocol & cell number / ChIP**	**Bench’**	**New**	**New**	**New**	**New**	**New**
	**2 x 10**^**7**^	**2 x 10**^**7**^	**2.5 x 10**^**6**^	**5 x 10**^**5**^	**1 x 10**^**5**^	**2 x 10**^**4**^
Total number reads	13 402 262	13 798 839	14 136 243	16 693 519	15 601 563	56 904 707
Number unique, non-duplicate reads	6 011 891	7 0330 709	5 794 519	3 463 886	2 423 126	661 591
Number of peaks called (fraction relative to benchmark)	16 545	16 244	17 054	15 636	14 771	12 296
	(1)	(0.98)	(1.03)	(0.95)	(0.89)	(0.74)
Sensitivity relative to benchmark	1	0.93	0.96	0.89	0.85	0.69
Specificity relative to benchmark	1	0.94	0.93	0.95	0.97	0.98

Peaks were called using MACS, allowing no ambiguously mapping or duplicate reads.

Using the method of Zhao and colleagues as a benchmark, the overlap with peaks called from the new method was evaluated as a measure of sensitivity (Table 1). Sensitivity was well maintained down to 1 × 10^5^ cells / IP, where 85% of peaks could still be detected. As expected from the reduced number of peaks called, sensitivity fell in the lowest cell number sample to 70%. Peaks were not lost randomly with reduced cell numbers. Rather, the same peaks were affected in each sample, with the preferential loss of those with lowest significance (fewest reads) evident as cell numbers were reduced.

Importantly, peak position was not adversely affected by lowering cell number. As can be seen in Figure 3b, peak overlaps between the benchmark and other datasets were close to 100% (i.e. not partially overlapping, or within an arbitrarily chosen window size). However, peak width was reduced in the lowest cell number samples (Figure 3c). Whilst every effort was made to ensure similar MNase digestion between the samples, we cannot exclude that the narrower peaks seen with lower cell numbers are due to increased digestion in these samples. However, the effect may be entirely explained by the lower number of reads available for peak calling in these samples. We are currently unable to separate the two possibilities.

In addition, extra peaks not present in the benchmark dataset were used to calculate a measure of specificity (Table 1). Specificity was not affected by scaling down cell numbers, with all datasets having greater than 90% of called peaks “on target” when comparing to the benchmark. It should be noted that this assumes that the dataset of Zhao and colleagues represents a gold standard and that additional peaks are false positives, which is not necessarily the case. Comparing the signal intensity of peaks at each location revealed a strong correlation, which deteriorated at the lowest cell number of 2 × 10^4^ cells per IP (Figure 3d).

**Figure 4:**
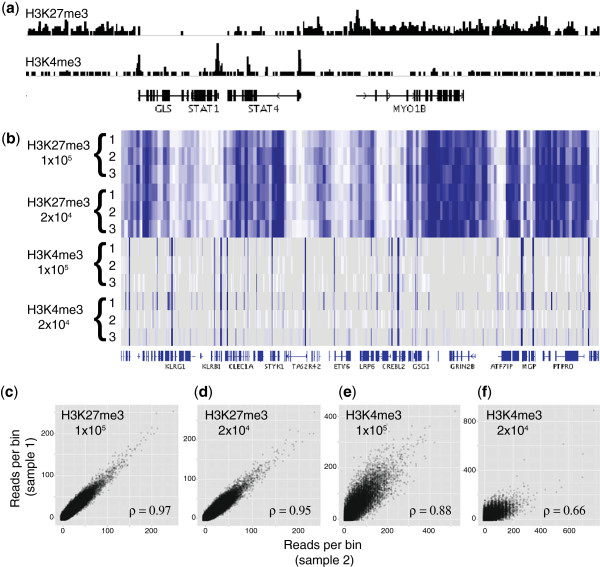
**Reproducibility of H3K4me3 and H3K27me3 ChIP-seq with the new method.** (**a**) 1Mb region of chromosome 2 containing the transcriptionally active *STAT1* / *4* and inactive *MYO1B* loci, visualised in the IGV genome browser. (**b**) Heatmap display in IGV genome browser showing triplicate ChIP signals over an 8 Mb region on chromosome 12. H3K27me3 and H3K4me3 signals are shown for 50 and 10 kb window sizes respectively. (**c**-**f**) Genome-wide pairwise correlations of read depth in 50 kb (H3K27me3) and 10 kb (H3K4me3) bins for selected replicate samples. Pearson correlation coefficients are given for each comparison. Read depth per bin was normalised to the total number of uniquely mapping reads per sample (reads per bin per million uniquely mapped reads).

To demonstrate the application of the new method to other histone modifications, the transcriptionally repressive H3K27me3 mark was also examined. Figure 4a shows the ChIP-seq profile of H3K4me3 and H3K27me3 generated from 100,000 cells / IP at the active *STAT4* and inactive *MYO1B* loci (a comparable image can be found in the paper of Barski et al., [20]). The mutually exclusive nature of H3K4 and H3K27 trimethylation is clearly visible in these profiles.

To assess the reproducibility of data generated with the new method, three independent chromatin extracts were prepared from frozen primary CD4^+^ lymphocytes, from which H3K4me3 and H3K27me3 ChIP-seq profiles were generated using 100,000 and 20,000 cells per IP. A heatmap display allowing comparison of the read depths across all 12 samples is presented in Figure 4b, and shows the similarity of the replicate samples across an 8 Mb chromosomal section. The mutually-exclusive nature of the two histone modifications is also clearly visible at this scale. A quantitative measure of pairwise ChIP signal intensities (Pearson′s correlation coefficient) is shown for selected replicate pairs in Figure 4c-f, and a complete matrix of correlation coefficients is provided as Table 2. Correlation coefficients are high for both H3K4me3 and H3K27me3 datasets from 100,000 cells/ IP, and drop when 20,000 cells were used, supporting our earlier observations that the sensitivity of the technique is adversely affected at this low level of input material. The mutually exclusive nature of H3K4 and H3K27 trimethylation is confirmed here by the negative correlations seen when comparing these datasets. Four ChIP-seq datasets from the ENCODE project [27] have been included for comparison (two replicates each of H3K4me3 and H3K27me3 from the lymphoblastoid cell line GM12878 [28]), and show similar or poorer correlation coefficients than the datasets generated here from 100,000 cells / IP (Table 2).

**Table 2:** Genome-wide pairwise correlation coefficients of replicate ChIP experiments

	**GM**	**GM**	**GM**	**GM**	**H3**	**H3**	**H3**	**H3**	**H3**	**H3**	**H3**	**H3**	**H3**	**H3**	**H3**	**H3**
	**H3**	**H3**	**H3**	**H3**	**K27**	**K27**	**K27**	**K27**	**K27**	**K27**	**K4**	**K4**	**K4**	**K4**	**K4**	**K4**
	**K27**	**K27**	**K4**	**K4**	**me3**	**me3**	**me3**	**me3**	**me3**	**me3**	**me3**	**me3**	**me3**	**me3**	**me3**	**me3**
	**me3**	**me3**	**me3**	**me3**	**100k**	**100k**	**100k**	**20k**	**20k**	**20k**	**100k**	**100k**	**100k**	**20k**	**20k**	**20k**
	**R1**	**R2**	**R1**	**R2**	**R1**	**R2**	**R3**	**R1**	**R2**	**R3**	**R1**	**R2**	**R3**	**R1**	**R2**	**R3**
GM H3K27 me3 R1	1.00	0.86	0.07	−0.02	0.70	0.70	0.70	0.70	0.69	0.69	−0.05	−0.05	−0.05	0.01	0.02	0.06
GM H3K27 me3 R2	0.86	1.00	0.17	0.16	0.74	0.70	0.72	0.72	0.73	0.70	0.04	0.05	0.05	0.09	0.10	0.12
GM H3K4 me3 R1	0.07	0.17	1.00	0.95	0.01	0.02	0.01	0.02	0.02	0.03	0.68	0.67	0.69	0.57	0.63	0.51
GM H3K4 me3 R2	−0.02	0.16	0.95	1.00	0.00	−0.01	−0.01	0.00	0.01	0.00	0.72	0.71	0.74	0.59	0.64	0.49
H3K27me3 100k R1	0.70	0.74	0.01	0.00	1.00	0.95	0.97	0.96	0.96	0.94	−0.09	−0.09	−0.09	−0.06	−0.06	−0.03
H3K27me3 100k R2	0.70	0.70	0.02	−0.01	0.95	1.00	0.94	0.94	0.93	0.92	−0.09	−0.09	−0.09	−0.05	−0.05	−0.01
H3K27me3 100k R3	0.70	0.72	0.01	−0.01	0.97	0.94	1.00	0.95	0.95	0.93	−0.09	−0.09	−0.09	−0.05	−0.05	−0.02
H3K27me3 20k R1	0.70	0.72	0.02	0.00	0.96	0.94	0.95	1.00	0.95	0.93	−0.09	−0.09	−0.09	−0.05	−0.05	−0.02
H3K27me3 20k R2	0.69	0.73	0.02	0.01	0.96	0.93	0.95	0.95	1.00	0.93	−0.08	−0.08	−0.08	−0.04	−0.04	−0.01
H3K27me3 20k R3	0.69	0.70	0.03	0.00	0.94	0.92	0.93	0.93	0.93	1.00	−0.08	−0.09	−0.09	−0.04	−0.04	0.00
H3K4me3 100k R1	−0.05	0.04	0.68	0.72	−0.09	−0.09	−0.09	−0.09	−0.08	−0.08	1.00	0.86	0.88	0.70	0.76	0.59
H3K4me3 100k R2	−0.05	0.05	0.67	0.71	−0.09	−0.09	−0.09	−0.09	−0.08	−0.09	0.86	1.00	0.87	0.69	0.76	0.59
H3K4me3 100k R3	−0.05	0.05	0.69	0.74	−0.09	−0.09	−0.09	−0.09	−0.08	−0.09	0.88	0.87	1.00	0.70	0.77	0.58
H3K4me3 20k R1	0.01	0.09	0.57	0.59	−0.06	−0.05	−0.05	−0.05	−0.04	−0.04	0.70	0.69	0.70	1.00	0.66	0.56
H3K4me3 20k R2	0.02	0.10	0.63	0.64	−0.06	−0.05	−0.05	−0.05	−0.04	−0.04	0.76	0.76	0.77	0.66	1.00	0.60
H3K4me3 20k R3	0.06	0.12	0.51	0.49	−0.03	−0.01	−0.02	−0.02	−0.01	0.00	0.59	0.59	0.58	0.56	0.60	1.00

Pearson′s correlation coefficients for all pairwise sample comparisons were calculated for read depth across the genome divided into 50 kb (H3K27me3) or 10 kb (H3K4me3) non-overlapping bins. Replicate datasets derived from 100,000 cells / IP (100k) and those from 20,000 cells / IP (20k) are denoted by suffixes R1- R3. For comparison, four ENCODE datasets (two replicates each of H3K27me3 and H3K4me3) from the cell line GM12878, a lymphoblastoid cell line, have been included (GM; replicates denoted by R1 and R2).

Finally, to demonstrate the utility of the method using primary cell isolates, we applied it to cell samples from three pairs of human monozygotic twins. Using purified and live-frozen CD4^+^ and CD8^+^ lymphocytes (in the range of 365–500,000 cells per IP), H3K4 trimethylation profiles were prepared from each individual. A rudimentary comparison of peak calling in the three twin pairs is included here to demonstrate application of the method to primary cell isolates (Table 3). Peaks were considered concordant between a twin pair if peaks called in both individuals overlapped. Peak concordance ranged from 82–94% in CD4^+^ cells and 73–78% in CD8^+^ lymphocytes. A more complete analysis of several twin pairs, aimed at identifying differential methylation between twins, is outside the scope of this manuscript and will be presented elsewhere.

**Table 3:** ChIP-seq from primary cells isolated from human monozygotic twins

**Twin Pair**	**Twin pair 1**		**Twin pair 2**		**Twin pair 3**	
**CD4**^**+**^**cells**						
Cell no. / IP	5 x 10^5^	5 x 10^5^	5 x 10^5^	5 x 10^5^	5 x 10^5^	5 x 10^5^
No. reads	35 244 517	44 255 574	40 644 508	45 738 891	38 332 819	29 484 478
No. unique, nonduplicate reads	3 978 339	6 524 094	3 316 312	3 340 926	3 130 804	1 167 824
No. Peaks called	14 828	12 622	15 598	15 731	15 825	13 719
No. overlapping peaks (%)	12 457 (82%)		14 833 (94%)		13 091 (83%)	
**CD8**^**+**^**cells**						
Cell no. / IP	4.3 x 10^5^	3.7 x 10^5^	4.7 x 10^5^	5 x 10^5^	3.8 x 10^5^	4.2 x 10^5^
No. reads	45 245 361	35 681 254	38 455 042	35 257 788	34 505 357	41 729 689
No. unique, nonduplicate reads	18 996 309	4 778 486	12 403 566	6 654 203	5 840 308	4 438 574
No. Peaks called	17 704	18 720	20 728	18 145	18 743	20 899
No. overlapping peaks (%)	14 578 (78%)		18 828 (76%)		15 312 (73%)	

Peaks were called using MACS, allowing no ambiguously mapping or duplicate reads. Peaks with p-values > 1x10^-10^ were excluded from analysis. Numbers of overlapping peaks were counted, and expressed as a percentage of the highest peak count for the twin pair.

## Discussion

In this study we have developed and employed a rapid N-ChIP technique applicable to small cell numbers, which functions well down to 100,000 cells / IP. Whilst this limit is higher than the requirements reported using alternative HTS library preparation methods [11-13], it minimizes the use of nucleic acid amplification and associated risk of bias in the data. As the first N-ChIP protocol tailored to low cell numbers, it therefore offers an attractive alternative method to map the genome-wide distribution of histone modifications. The success of the method using standard HTS library preparation techniques may reflect the reported higher efficiency of N-chip relative to X-ChIP [15]. We would also like to stress the importance of selecting specific antisera, such as using a peptide array as employed here, to the success of this or any ChIP protocol using low input cell numbers. We have demonstrated the application of this method to immuno-purified CD4^+^ and CD8^+^ primary lymphocytes, thus avoiding the need for cell culture, which risks altering epigenetic modifications. Nonetheless, the study highlights the need for careful monitoring of sequence read mapping in the analysis of ChIP-seq data from limited cell numbers, to identify sources of wasted reads and ensure sufficient coverage for reliable peak calling.

As demonstrated here, PCR amplification from limited ChIP input material led to a reduction of mappable and unique reads through losses to amplification artifacts and duplicate molecules, which must be removed for reliable peak calling. The accumulation of such undesirable amplification artifacts in alternative techniques such as LinDA and nano-ChIP-seq [11-13] has not been adequately addressed to date, and requires further examination. Notably, the method presented here entails fewer amplification cycles than these other techniques. The introduction of bias by genome-amplification techniques such as linker-mediated PCR and WGA have been documented [29]. It is therefore desirable to minimise the number of amplification cycles whenever possible. It is likely that the levels of PCR artifacts could be reduced by employing alternative amplification conditions, or through the use of alternative polymerases.

It has been shown that both MNase digestion and sonication demonstrate some DNA cleavage sequence preference, which necessitates the use of control digestion / sonication of naked DNA when interpreting HTS data with the aim of identifying nucleosome positions or regions of high accessibility such as promoters [30,31]. The existence of favourable cleavage sites will inevitably lead to a number of “duplicate” reads that are in fact biological in origin rather than amplification artifacts. This effect is likely to be more pronounced in MNase digested chromatin, as the preferential digestion of MNase in inter-nucleosomal linker regions further restricts genomic cleavage sites. It is therefore possible that the levels of duplication seen here will not be so high when using formaldehyde cross-linked chromatin, although this remains to be tested. Using the data presented here, inclusion of duplicate reads led to the appearance of many non-specific peaks, so it remains challenging to differentiate genuine biological duplicate reads from those arising as the result of the PCR process. The percentage of duplicate reads derived from PCR could be further reduced by employing paired-end reads rather than the single reads typically used, as here, for ChIP-seq analysis. Only in the situation where molecules have exactly the same length and genomic position, would paired reads be counted as a duplicate. Alternatively, utilizing multiple combinations of indexed adapters, termed digital sequencing [32], would allow the differentiation of biological and PCR-derived duplicate reads.

In addition to the problems of amplification artifacts and duplicate reads, ChIP-seq from limiting cell numbers is challenging due to the reduced complexity of immunoprecipitated material recovered. In the case of CD4^+^ cells presented here, sensitivity and reproducibility were not badly affected down to 100,000 cells / IP, but below this were reduced, although useful results were nonetheless obtained (70% of peaks detected by benchmark method). Further improvements are likely possible by increasing the efficiency of immunoprecipitation, DNA purification, and sequencing library generation methods.

The future adoption of epigenetic analyses in research and diagnostic procedures will require techniques that allow analysis of specific cell types, sub-populations and small biopsy samples. For example, the N-ChIP technique has been optimised for analysis of the *Schistosoma* parasite [33], and techniques have been developed for ChIP from particular structures such as the hippocampus [34]. We have demonstrated here the application of the enhanced method to lymphocytes isolated from human twins. The cells used for study were enriched using antibody-driven magnetic cell sorting, derived from a starting material of 10–20 mls blood. It would not have been possible to study such small, easily obtained biological samples using previously published N-ChIP procedures. Further improvements in both ChIP protocols and HTS library preparation methods, such as single molecule sequencing [35] promise to increase the possibilities for epigenetic studies from these and other challenging samples.

## Conclusions

Using an enhanced native ChIP-seq method, we have detailed known but hitherto uncharacterised problems of performing ChIP-seq from limited cell numbers. Using our method, high quality results were obtained from 200,000 cells starting material (using 100,000 cells per IP), increasing possibilities for the study of rare cell populations and biopsy samples without the need for cell culture. It was possible to generate results from as low as 20,000 cells per IP, but at a cost to sensitivity, where only 70% of known peaks could be detected, so we declare the limit of this method to currently require 100,000 cells per IP. This method offers an alternative to other previously published methods for low cell number ChIP-seq that entails fewer cycles of amplification with associated risk of data bias. Nonetheless, as cell numbers are reduced, the amount and complexity of immunoprecipitated material is reduced. Amplification of this material leads to a corresponding rise in PCR duplicates and unmapped reads, which may necessitate costly additional sequencing, an effect that requires further characterisation in comparable methods. Additional improvements in the efficiency of immunoprecipitation and HTS library generation techniques are desirable to bring us closer to the goal of single cell analysis.

## Methods

### Isolation of lymphocyte sub-populations & cell culture

Peripheral blood mononuclear cells (PBMCs) were isolated from an anonymous human blood donor using Lymphoprep reagent (Axis-Shield plc, Dundee, UK). CD8^+^ and CD4^+^ cells were sequentially isolated using an AutoMACS Pro separator (Miltenyi Biotec, Köln, Germany) by positive and negative isolation, respectively. Cells were live-frozen in freezing medium (20% v/v DMSO; 80% v/v Fetal Calf Serum (Lonza, Basel, Switzerland)) in aliquots. For experiments requiring up to 4 × 10^7^ cells per chromatin preparation, two million thawed CD4^+^ cells were activated using Human T-Expander CD3/CD28 (Invitrogen, Carlsbad, CA) according to manufacturers instructions and cells were cultured in X-VIVO media (Lonza) supplemented with Interleukin II (10 ng/ul) for a period of two weeks at 37°C in a humidified incubator under 5% CO_2_. For experiments with human twin cell populations, thawed CD4^+^ or CD8^+^ cells were used directly as input into the ChIP procedure as detailed below.

### Antibody selection

Polyclonal antisera against H3K4me3 (Diagenode s.a., Liège, Belgium; Cat. # pAb-003-050; Lot # A2-002P) and H3K27me3 (Upstate Biotechnology, Temecula, CA; Cat. # 07-449; Lot # DAM1387952) were selected after screening several commercially available antisera for specificity using a custom histone peptide microarray (JPT Peptide Technologies Gmbh, Berlin, Germany). Arrays were spotted with 156 different selected peptides of length 13 residues, representing covalently modified N-terminal tails of H3 and H4 (details available upon request). Results of specificity testing are available in the HisMAD database [36].

### Chromatin immunoprecipitation

The method of Zhao and colleagues [21] was followed and used as a benchmark to which our new method was compared. The new method differs from the benchmark in the following ways: (i) The requirement for dialysis was removed by instead diluting chromatin in a concentrated immunoprecipitation buffer, allowing faster handling and reduced material loss. (ii) Sonication was performed in a Bioruptor sonicator (Diagenode, Liège, Belgium) using TPX plasticware to allow sonication of small volumes with minimal sample loss and eliminate potential sources of contamination. (iii) Control of MNase Digestion was performed on a 2100 Bioanalyzer (Agilent Technologies, Santa Clara, CA) to allow visualisation of much lower DNA amounts. (iv) Illumina library preparation was altered to retain nucleosome-sized fragments upon size selection, and (v) replace column-based cleanup steps with SPRI-beads (Beckman Coulter, Beverly, MA) to retain more DNA at every step.

The method is summarised below. In addition, a detailed step-by-step method is provided as Additional file 1. The method presented here is specifically tailored for sequencing on Illumina technology, but should be easily adaptable to other sequencing platforms. Cultured CD4^+^ cells were counted, then harvested by centrifugation at 1500 × G for 5 mins at room temp and washed in PBS. Cells were divided at this stage into separate tubes according to the titration of starting cell numbers required. Cell pellets were then resuspended in digestion buffer (50 mM Tris–HCl, pH 8.0; 1 mM CaCl_2_; 0.2% Triton X-100) at room temperature supplemented with protease inhibitors. Because of impracticalities associated with handling cell numbers spanning 3 orders of magnitude, it was not possible to use exactly the same volumes and concentrations for all steps, and these differences have been summarised in Table 4. Micrococcal nuclease (USB, Cleveland, OH) was added to a concentration of 0.19 units per 1 × 10^7^ cells, and incubated for 5 mins at 37°C. Digestion was terminated by the addition of 0.1 volumes of stop solution (110 mM Tris pH 8.0; 55 mM EDTA) and samples transferred to ice. Samples were then subjected to brief sonication in a Bioruptor (Diagenode) for 60 seconds, on high power with no pulsing in TPX tubes (Diagenode) to assist with recovery of oligonucleosomes. Samples were then adjusted to RIPA buffer conditions by the addition of 1 volume 2xRIPA IP buffer (280 mM NaCl; 1.8% Triton X-100; 0.2% SDS; 0.2% Na-Deoxycholate; 5 mM EGTA) supplemented with protease inhibitors, and spun in a microcentrifuge at 16,000 × G for 15 mins at 4°C. Supernatants were immediately removed to fresh tubes and 10% volume removed for DNA purification as “input”. DNA was later purified from input samples by adding proteinase K (USB, Cleveland, OH) to a final concentration of 0.5 mg/ml and incubating at 55°C for 1 hour, prior to purification over a Genomic DNA cleanup & Concentrator column (Zymo Research Corp., Irvine, CA). The size of input DNA was then measured on a 2100 Bioanalyzer (Agilent Technologies) using high sensitivity reagents, to check that all samples had similar digestion levels and were primarily composed of mono-nucleosomes. The remaining majority of the chromatin was immediately used for immunoprecipitation. Chromatin was first pre-cleared by the addition of a 1:1 mix of protein A and G Dynabeads (Invitrogen, Carlsbad, CA) with rotation at 4°C for 1 hour (see Table 4). Chromatin was then divided into two for specific IP with H3K4me3 or control IgG (see Table 4) and incubated overnight with rotation, followed by immunoprecipitation with protein A and G Dynabeads for 2 hours at 4°C. Beads were immobilised on magnetic racks and the supernatants discarded, after which the beads were washed five times with RIPA buffer (10 mM Tris pH 8.0; 1 mM EDTA; 140 mM NaCl; 1% Triton X-100; 0.1% SDS; 0.1% Na-Deoxycholate) and once with LiCl wash buffer (250 mM LiCl; 10 mM Tris pH 8.0; 1 mM EDTA; 0.5% Igepal CA-630; 0.5% Na-deoxycholate). All washes were carried out for 5 mins at 4°C on a rotating wheel (see Table 4 for volumes) in the presence of protease inhibitors. Beads were finally rinsed with TE buffer without protease inhibitors. Beads were then resuspended in 100 μl TE containing 0.5 mg/ml proteinase K and incubated at 55°C for 1 hour with shaking, prior to purification over a purification column as detailed for input DNA above, eluting in 50 μl 5 mM Tris buffer.

**Table 4:** Variable parameters applied to chromatin from different starting cell numbers

**Protocol & starting cell number**	**Benchmark**	**New**	**New**	**New**	**New**	**New**
	**4 x 10**^**7**^	**4 x 10**^**7**^	**5 x 10**^**6**^	**1 x 10**^**6**^	**2 x 10**^**5**^	**4 x 10**^**4**^
Cells per IP	2 x 10^7^	2 x 10^7^	2.5 x 10^6^	5 x 10^5^	1 x 10^5^	2 x 10^4^
MNase digestion volume (cells / ml)	1500 μl	1500 μl	500 μl	100 μl	20 μl	20 μl
	(2.7 x 10^7^/ml)	(2.7 x 10^7^/ml)	(1 x 10^7^ / ml)	(1 x 10^7^ / ml)	(1 x 10^7^ / ml)	(2 x 10^6^ / ml)
IP volume	750 μl	1500 μl	500 μl	100 μl	100 μl	100 μl
	(in 1.5 ml tube)	(in 2 ml tube)	(in 1.5 ml tube)	(in 0.2 ml PCR tube)	(in 0.2 ml PCR tube)	(in 0.2 ml PCR tube)
Protein A/G bead volume for preclearing / IP	50 μl	50 μl	50 μl	10 μl	10 μl	10 μl
Antibody amount / IP	5μg	5μg	5μg	1 μg	1 μg	1 μg
Wash buffer volumes	1 ml	1 ml	1 ml	150 μl	150 μl	150 μl

“Benchmark” refers to the protocol now published by Zhao and colleagues [21] and “new” to the method presented here.

### Real-time PCR

Two microliters of ChIP eluate were used per reaction to control the success of the immunoprecipitations using the following real-time PCR primer combinations: H3K4me3 positive control locus TaqMan assay (probe with 5^′^ FAM and 3^′^ blackhole quencher 1) [RPL30-F CAAGGCAAAGCGAAATTGGT; RPL30-R GCCCGTTCAGTCTCTTCGATT; RPL30-P TCTCGCTAACAACTGCCCAGCTTTGAG], negative control locus SYBR Green assay [NegC1-F ACGTACCTTAAGCCCCTGGT; NegC1-R TAGTGCCTGGAGTGAGGATG]. Primers were obtained from MWG Biotech (Ebersberg, Germany), and reactions performed using TaqMan or SYBR Green universal PCR master mixes (Applied Biosystems, Foster City, CA).

### Illumina library preparation

Twenty five microliters of immunoprecipitated or input DNA (ranging from <1 ng – 4 ng DNA) was used for library preparation using Illumina (San Diego, CA) TruSeq™ DNA Sample Preparation reagents, with ligation of 1/10^th^ the manufacturers recommended adapter amounts and agarose gel selection of DNA fragments in the 200–500 bp size range.

### High-throughput sequencing

Sequencing (36 bp single reads) was performed on an Illumina Genome Analyzer IIx using v4 cluster generation and v3 sequencing reagents, using one lane per sample. Yields varied within the range of 13.4 to 17.1 million reads passing filters per sample (0.48 – 0.62 Gb). Alternatively, for additional sequencing of a single sample (ChIP from 2 × 10^4^ cells) and for all ChIP experiments using live-frozen human lymphocytes, libraries were sequenced (50 bp single-end reads) on an Illumina HiSeq 2000 using TruSeq v2.0 clustering and SBS sequencing reagents. Samples run on the HiSeq 2000 were indexed and run together, such that each sample obtained reads equivalent to half a lane on the flow cell.

### Data analysis

Illumina GAIIx image analysis and base calling was performed using Illumina’s RTA software version 1.4 and experiments performed on the Illumina HiSeq 2000 were analysed using RTA version 1.12. Reads were filtered to remove those with low base call quality using Illumina’s default chastity criteria. Reads were mapped to the human reference genome (release hg18 / NCBI36) using BWA version 0.5.9 with default settings [37]. Peak calling was performed by MACS version 1.4 [26] using shift size determined by the size of sequencing library inserts, and switching off local background estimation (as recommended for histone modification peak calling in the absence of a control immunoprecipitation). Peak calling was only based on reads mapping to a single location, excluding duplicates. Manipulation of alignment and peak files was performed using samtools version 0.1.15 [38] and bedtools version 2.11.2 [39] software. We investigated the reproducibility of the protocol between different samples by dividing the genome into non-overlapping 10k or 50k bins and then computing the number of uniquely mapping non-duplicate reads in each bin. The count of reads in each bin was then normalised for the sample’s total number of uniquely mapping non-duplicate reads. In order to visualise the same data in a genome browser, we applied the count command of the igvtools utilities to the dataset of uniquely mapping non-duplicate reads and displayed the result in IGV using the heatmap setting [25].

### Histone methylation levels surrounding gene start positions

Gene expression data for CD4^+^ T lymphocytes was obtained from the Gene Expression Omnibus [40] dataset GSE473 [41]. Affymetrix microarray design U133A expression data was retrieved for 10 control individuals, from which average gene expression levels were calculated and used to assign genes to highest or lowest quartile expression categories. H3K4me3 ChIP read depth was calculated 2 kb upstream and downstream of gene start positions.

## Abbreviations

HTS: High throughput sequencing; IP: Immunoprecipitation; ChIP: Chromatin immunoprecipitation; N-ChIP: Native chromatin immunoprecipitation; X-ChIP: Formaldehyde cross-linked chromatin immunoprecipitation; ChIP-seq: Genome-wide analysis of ChIP employing high throughput sequencing; H3K4me3: Histone H3 lysine 4 trimethylation; MNase: Micrococcal nuclease; TSS: Transcription start site.

## Competing interests

The authors declare no competing interests.

## Authors’ contributions

The study was designed by GDG as part of a project conceived by RL, DEU and GDG. ChIP and sequencing library preparation was performed by HSH and GDG. Donor cell samples were assimilated by JH, and cell isolation procedures developed by KG. Antibody specificity testing was performed by TS. Data analysis was performed by TH and YS, and interpreted by TS and GDG. All authors contributed to the manuscript and approved it for publication.

## Supplementary Material

Additional file 1 A detailed protocol of the native ChIP-seq procedure described herein. Click here for file
