# Supplementary material for: Limitations and possibilities of low cell number ChIP-seq
Source: BMC Genomics. 2012 Nov 21;13:645. doi: 10.1186/1471-2164-13-645 (PMC3533509; doi:10.1186/1471-2164-13-645)
Supplement: Additional file 1 — A detailed protocol of the native ChIP-seq procedure described herein. [file 1471-2164-13-645-S1.pdf]

Low cell-number Native Chromatin Immunoprecipitation-sequencing (ChIP-seq) Protocol

Supplementary method: Gilfillan et al.

The protocol below is written for frozen, live cells, but can easily be adapted for use with freshly prepared samples. As written below, it has been optimised for sorted $CD4^+$ & $CD8^+$ lymphocytes in the range of $2 \times 10^5 – 1 \times 10^6$ cells (starting cell numbers). For lower cell numbers, scaling all volumes down by a factor of 5 has been tested and works well down to at least 4×10^4 cells. The protocol generates chromatin than can be diluted and used for multiple immunoprecipitations (e.g. chromatin equivalent to 2×10^4 cells per IP). In particular, it is likely that each batch of MNase purchased may require optimisation to adjust for differences in activity, so it is recommended to purchase sufficient MNase for multiple experiments and freeze this in appropriate aliquots.

MATERIALS REQUIRED (standard laboratory suppliers unless specified)

1. Micrococcal Nuclease (MNase): e.g. USB product #70196Y.
   -Resuspend in 50% glycerol at 1 mg / ml (1.15 Units / µl) , store at -20 ^oC **= 1x stock**
   -Diluted 1 /10 with further 50% glycerol, store at -20 ^oC **= 0.1x stock** (allows use of more accurate pipetting volumes due to the small amounts required in this protocol)
2. Proteinase K (e.g. USB product #76230Y)
   -Dissolve in H_2O to 10 mg / ml and store at -20 ^oC
3. Specific antibodies (ChIP-seq tested) and control IgG (e.g. #sc-2027; Santa Cruz Biotech, Santa Cruz, CA)
4. TPX 1.5 ml tubes (Diagenode s.a., Liège, Belgium)
5. 8-well 0.2ml strip tubes (e.g. from Applied Biosystems, Foster City, CA)
6. Protein A Dynabeads (Invitrogen, Carlsbad, CA)
7. Protein G Dynabeads (Invitrogen, Carlsbad, CA)
8. DNA purification columns (e.g. Genomic DNA Clean & Concentrator; Zymo Research, Irvine, CA)
9. Protease Inhibitor Cocktail (e.g. P8340; Sigma-Aldrich, St. Louis, MO). Use at 100x dilution (high conc.) or 1000x dilution (low conc.)
10. Reagents & primers for quantitative PCR
11. General laboratory chemicals required for buffer preparation: 1M Tris-HCl pH 8.0, 0.5M EDTA, 1M $CaCl_2$, 5M NaCl, 10% SDS, 10% Sodium deoxycholate, 0.5M EGTA, Triton X-100, Igepal CA-630, KCl, Na_2HPO_4, KH_2PO_4.
12. Illumina TruSeq™ DNA sample preparation kit (part number FC-121-1001; Illumina, San Diego, CA)
13. Agencourt AMPure XP DNA purification beads (Beckman Coulter, Beverly, MA)
14. 99% pure laboratory ethanol

15. Materials for TAE gel electrophoresis, including scalpel for band excision, DNA size standards and ethidium bromide for staining. Recommended agarose = Certified Low Range Ultra Agarose (Biorad, Hercules, CA)
16. MinElute gel extraction kit (product # 28604; Qiagen, Hilden, Germany)
17. Qubit dsDNA HS Assay Kit (product #Q32851, Invitrogen, Carlsbad, CA)
18. 2100 Bioanalyzer High Sensitivity DNA Kit (product #5067-4626; Agilent Technologies, Santa Clara, CA)

EQUIPMENT REQUIRED (standard laboratory suppliers unless specified)

1. Cold Room
2. Magnet for 1.5 ml tubes (e.g. DynaMag-2; Invitrogen, Carlsbad, CA)
3. Magnet for 0.2 ml tubes (e.g. DiaMag02; Diagenode s.a., Liège, Belgium)
4. Bioruptor (Diagenode s.a., Liège, Belgium)
5. 2100 Bioanalyzer (Agilent Technologies, Santa Clara, CA)
6. Microfuge for 1.5 ml tubes - room temp
7. Microfuge for 1.5 ml tubes - chilled
8. Mini centrifuge with rotor for 8-well strips
9. Rotating wheel with mountings holding 8-well strips
10. Shaking thermal block for 1.5 ml tubes
11. Quantitative real-time PCR machine
12. (Optional) Aspirator apparatus & waste trap
13. Equipment for agarose gel electrophoresis
14. Dark reader transilluminator (product #D195M; Clare Chemical Research, Dolores CO)
15. 2100 Bioanalyzer (Agilent Technologies, Santa Clara, CA)
16. Qubit Fluorometer (Invitrogen, Carlsbad, CA)

BUFFERS

1. MNase Digestion buffer

- 50 mM Tris-HCl, pH 8.0
- 1 mM $CaCl_2$
- 0.2 % Triton X-100

2. 10x MNase Stop buffer

- 110 mM Tris-HCl pH 8.0
- 55 mM EDTA

3. 10x PBS

Dissolve the following in H_2O, then autoclave. Prepare a fresh 1x stock from this prior to starting protocol below.

- 80 g NaCl
- 2 g KCl
- 14.4 g Na_2HPO_4
- 2.4 g KH_2PO_4

4. **2x RIPA IP buffer (on ice)**

- 280 mM NaCl
- 1.8 % Triton X-100
- 0.2 % SDS
- 0.2 % Na-Deoxycholate
- 5 mM EGTA

5. **RIPA buffer (on ice)**

- 10 mM Tris pH 8.0
- 1 mM EDTA
- 140 mM NaCl
- 1 % Triton X-100
- 0.1% SDS
- 0.1 % Na-Deoxycholate

6. **LiCl wash buffer (on ice)**

- 250 mM LiCl
- 10 mM Tris pH 8.0
- 1 mM EDTA
- 0.5 % NP-40 (now known as Igepal CA-630)
- 0.5 % Na-deoxycholate

7. **TE (on ice)**

- 10 mM Tris-HCl pH 8.0
- 1 mM EDTA

Notes on buffer preparation:

Triton X-100 should be added from a 10% (w/v) stock in H_2O: For greatest accuracy weigh the liquid triton (density 1.07 g / ml). Prepare Igepal Ca-630 in a similar manner.

Na-Deoxycholate 10% stock: Filter sterilise after dissolving in H_20.

DAY 1

1. ***Thaw & spin down cells in TPX tubes.***
   Thaw an aliquot of frozen cells in a water bath at 37°C. Immediately transfer the cells to a 1.5 ml TPX tube and centrifuge 5 mins, 1500 x G, room temp.
2. ***Wash cells with PBS.***
   Discard supernatant in hazardous waste, and resuspend cell pellet in 1 ml PBS (room temp). Centrifuge again as above.
3. ***Dilute MNase in digestion buffer to 1/300 original conc.***
   Whilst cells are centrifuging, prepare a fresh working dilution of MNase sufficient for all samples you will process. Use pipetting volumes that will be most reproducible (e.g. 5 µl

"0.1x MNase" plus 145 µl digestion buffer). Store diluted enzyme on ice. Discard remaining diluted enzyme after use.

4. ***Resuspend cells in digestion buffer + protease inhibitors.***
   Remove & discard supernatant, then resuspend cell pellet in 95 µl digestion buffer per 1 x 10^6 cells (room temp) supplemented with protease inhibitors (high conc). Immediately pipette cells gently up and down several times to prevent aggregation of lysed cell components.
5. ***MNase Digestion***
   For every 1 x 10^6 cells, add 5 µl diluted MNase to cells, flick tube to mix, then place in 37 ^{o}C block (no shaking) for EXACTLY 5 mins. Process multiple samples in series so that each receives 5 mins incubation. Note that the amount of MNase required will need to be determined empirically for each new batch of enzyme purchased or for new cell types.
6. ***Add appropriate volume 10x stop buffer*,** mix thoroughly but gently**. Keep samples on ice / coldroom from now on.**
7. ***Sonicate in Bioruptor***
   Sonicate in the Bioruptor for 60 secs (high power, no pulsing). Volume in individual 1.5 ml tubes must not exceed 300 µl.
8. ***Adjust buffer to RIPA conditions*** by adding cold "2x RIPA IP buffer" (110 µl per 1 x 10^6 cells) supplemented with protease inhibitors (high conc). Mix by flicking tube.
9. ***Spin tubes for 15 mins*** in a microfuge, top speed, 4 ^{o}C.
10. ***Remove supernatants to fresh pre-cooled tubes*** (1.5 ml regular polypropylene) and store on ice.
11. ***Remove input for later use in qPCR / control of digestion on Agilent Bioanalyzer.***
    Remove 10% volume of chromatin and store for later purification. When convenient, digest this aliquot by adding TE to 95 µl and 5 µl proteinase K (10 mg / ml stock). Incubate with shaking (1200 rpm or greater) in a 55 ^{o}C block for 1 hour. Purify over a spin column (recommended = Zymo research gDNA #D4010) and elute in 10 µl 5 mM Tris. Load 1 µl on Bioanalyzer HS chip as per manufactures instructions. Digestion should reveal a majority of mono-nucleosomes (ca. 140 bp) with di- and tri- nucleosome peaks also clearly visible. Store remainder for use as "input" samples in qPCR.
12. ***Prepare sufficient protein A & G Dynabeads***
    You will need 25 µl beads (1:1 mix of protein A and protein G – this can be mixed in advance) per IP = 10 µl for preclearing, 10µl for IP and ca. 5 µl excess. Pipette the desired amount into a 1.5 ml tube, and using the magnet, wash them twice with 1 ml RIPA buffer +protease inhibitors (low conc). Finally, resuspend them to their original volume in RIPA buffer + protease inhibitors (low conc).
13. ***Dilute chromatin to 100µl/ IP***
    IPs will later be performed in 0.25 ml thin-walled PCR tubes, which give best mixing upon rotation with 100-150 µl volumes. If performing >2 IPs, dilute your chromatin now to allow ca. 100µl per IP. Dilute with RIPA + protease inhibitors (low conc).
14. ***Preclear chromatin with beads***
    For every IP you will perform, add 10 µl washed dynabeads to the chromatin. e.g. if you will perform 4 separate IPs, add 40 µl beads. Incubate with rotation at 4 ^{o}C for 1 hour.

15. ***Bind antibodies overnight***

Remove beads on magnet, and transfer 100 µl chromatin per IP to a well of an 8-well thin-walled tube strip. Add antibodies (amounts must be determined empirically), seal with 8-well strip caps, and incubate overnight with rotation at 4 ^{o}C. Be sure to perform appropriate negative controls such as pre-immune or control IgG IPs in addition to specific immunoprecipitations.

DAY 2

1. ***IP with protein A/G beads***

   Spin down liquid from lids of tubes in mini centrifuge. Add 10 µl beads per IP and incubate a further 2-3 hours with rotation at 4 ^{o}C.

2. ***Wash beads***

   Prepare sufficient RIPA and LiCl wash buffer for all IPs, supplemented with protease inhibitors (low conc.). All washes with rotation at 4 ^{o}C, 5 mins duration.

Wash as follows:	5 washes with	150 µl RIPA buffer
	1 wash with	150 µl LiCl wash buffer

   Perform washes in cold room, using magnet to collect beads between washes. Beads stuck in tube lids should be collected by briefly spinning tube strips in mini centrifuge prior to placing strips on magnet. Use of an aspirator to remove buffers will greatly speed this step.

3. ***Rinse beads*** with a final wash of cold TE (**no protease inhibitors**), 150 µl.

   Note that beads do not adhere so well to tube walls in TE buffer, so use a pipette rather than aspirator to remove TE.

4. ***Digest proteins with proteinase K***

   Prepare sufficient TE buffer supplemented with proteinase K (10 mg / ml stock). You need 95 µl TE and 5 µl proteinase K per IP.

   Resuspend each bead pellet in 100 µl TE / proteinase K solution, transfer to a fresh 1.5 ml tube, and incubate with shaking (1200 rpm or greater) in a 55 ^{o}C block for 1 hour.

5. ***Purify Immunoprecipitated DNA*** using a cleanup column.

- Add binding buffer to beads / TE mix and vortex
- Place tube on magnet and wait until beads aggregate
- Transfer supernatant to cleanup column, and proceed as in manufacturers instructions
- Elute in 50 µl 5 mM Tris for maximum recovery. Wait 1 min after adding elution buffer before spinning

6. **Verify IPs worked by qPCR** (use 2µl per 10 µl PCR reaction). Be sure to leave at least 30µl eluate for ChIPseq library generation, so dilute an aliquot for Q-PCR analysis if necessary. Test at least one positive & negative control region appropriate to each antibody before proceeding to prepare Illumina sequencing libraries.

DAY 3

Illumina sample library preparation.

The following procedure contains minor modifications from the protocol described in Illumina's TruSeq DNA Sample Preparation Guide (part no. 15005180 Rev. A). Do not use the end repair, A-tailing or ligation control DNAs supplied in the Illumina TruSeq kit when preparing samples from ChIP DNA. Users should consult the above preparation guide for handling and storage instructions regarding the reagents supplied with the kit. For all reaction setups below, pipette the components in order and mix by pipetting up and down 10 times when all components have been added.

1. ***Perform End Repair***

   Set up the reaction below (on ice) for each ChIP sample, then incubate at 30°C for 30 mins.

ChIP DNA from step 5 above:	25 µl
Resuspension buffer (RSB):	5 µl
End Repair Mix (ERP):	20 µl
**Total:**	**50 µl**

2. ***Warm AMPure XP beads to room temperature and prepare sufficient 80% ethanol for washing (prepare fresh each day).***

3. ***Clean up reactions using AMPure XP beads*** *(all steps at room temperature)*

- Add 80 µl AMPure XP beads to each reaction, mix, and incubate for 15 mins.
- Place the mixture on a magnetic rack and allow to clear (15 mins).
- Remove and discard supernatant, and leave tube on magnet.
- Wash beads twice with 200 µl 80% ethanol (freshly prepared).
- Remove all traces of ethanol with a pipette, then let tube stand for 5 mins (still on magnet) to allow ethanol to evaporate.
- Remove tube from magnet and resuspend beads in 20 µl RSB, then incubate 2 mins on bench.
- Replace tube on magnet and allow to clear. Transfer 17.5 µl to a fresh tube.

4. ***Adenylate 3' ends***

   Set up the reaction below (on ice) for each ChIP sample, then incubate at 37°C for 30 mins.

DNA from step 3 above:	17.5 µl
A-tailing Mix (ATL):	12.5 µl
**Total:**	**30 µl**

5. ***Prepare index adapters***

   Whilst A-tailing reactions are incubating, thaw and dilute TruSeq DNA adapters 1:10 with H_20. Consider at this point which reactions will be run together (indexed) in a lane of sequencing and select appropriate adapters for each sample. Discard unused diluted adapter after use.

6. ***Ligate adapters***

   Set up the reaction below (on ice) for each ChIP sample, then incubate at 30°C for **10** mins.

Adenylated DNA from step 4 above:	30 µl
Resuspension buffer (RSB):	2.5 µl
Ligase Mix (LIG:	2.5 µl
1/10 Diluted adapter:	2.5 µl
**Total:**	**37.5 µl**

7. ***Stop ligation***

   Add 5 µl Stop Ligase Mix (STL), mix by pipetting, centrifuge briefly. Store on bench, but proceed immediately.

8. ***Clean up reactions using AMPure XP beads*** *(all steps at room temperature)*

- Add 42.5 µl AMPure XP beads to each reaction, mix, and incubate for 15 mins.
- Place the mixture on a magnetic rack and allow to clear (2-5 mins).
- Remove and discard supernatant, and leave tube on magnet.
- Wash beads twice with 200 µl 80% ethanol (freshly prepared).
- Remove all traces of ethanol with a pipette, then let tube stand for 5 mins (still on magnet) to allow ethanol to evaporate.
- Remove tube from magnet and resuspend beads in 52.5µl RSB, then incubate 2 mins on bench.
- Replace tube on magnet and allow to clear. Transfer 50 µl to a fresh tube.

9. ***Perform an additional AMPure XP bead cleanup***

- Add 50 µl AMPure XP beads to each reaction, mix, and incubate for 15 mins.
- Place the mixture on a magnetic rack and allow to clear (2-5 mins).
- Remove and discard supernatant, and leave tube on magnet.
- Wash beads twice with 200 µl 80% ethanol (freshly prepared).
- Remove all traces of ethanol with a pipette, then let tube stand for 5 mins (still on magnet) to allow ethanol to evaporate. **It is essential at this stage that no traces of ethanol remain.**
- Remove tube from magnet and resuspend beads in 22.5µl RSB, then incubate 2 mins on bench.
- Replace tube on magnet and allow to clear. Transfer 20 µl to a fresh tube.

10. ***Size select ligation products on a 2% TAE agarose gel***

    Prepare a 2% TAE agarose gel prestained with ethidium bromide, and run the entire product of step 9 above in a single lane, flanked by size standards appropriate to the size range you wish to purify. Run the gel for no longer than necessary (typically 45 mins at ca. 100V) to separate the desired size range. No DNA will be visible in the target lane. Excise as broad a size range as possible within the 400 mg limit of the Qiaquick purification that follows, unless you are concerned your input chromatin has not been adequately sheared in the first place. For example, we regularly excise DNA in the range 200-500 bp. Smaller ranges are possible if greater resolution is desired, but may reduce the complexity of the resulting library. DO NOT

cut fragments below 130 bp, where adapter dimer products run, which will require additional cleanup following PCR.

11. ***Purify selected DNA using MinElute gel extraction columns***
    Follow the instructions in the MinElute gel extraction kit. However, dissolve agarose slices at room temperature, not by heating. Add isopropanol as directed to buffer QG prior to column loading, and perform additional QG wash as directed. Wait as directed after adding buffer PE prior to centrifugation. Finally, elute the purified DNA from the column in 22.5 µl Qiagen buffer EB.

12. ***PCR amplify isolated DNA***
    Set up the reaction below (on ice) for each ChIP sample, then amplify with the conditions specified.

DNA from step 11 above:	20 µl
TruSeq PCR primer cocktail:	5 µl
TruSeq PCR master mix:	25 µl
**Total:**	**50 µl**

Amplification conditions:

98 ^{o}C, 30 s
98 ^{o}C, 10 s]
60 ^{o}C, 30 s] Repeat for a total of 16-18 cycles*
72 ^{o}C, 30 s]
72 ^{o}C, 5 mins

* Empirical optimisation of the number of cycles required will be necessary, and is dependent on antibody specificity & affinity, the abundance of the targeted protein bound to chromatin, and cell type. The lowest suitable number of cycles is recommended to minimise PCR-induced bias and duplicate reads.

DAY 4

1. Clean PCR products using AMPure XP beads

- Add 50 µl AMPure XP beads to each reaction, mix, and incubate for 15 mins.
- Place the mixture on a magnetic rack and allow to clear (2-5 mins).
- Remove and discard supernatant, and leave tube on magnet.
- Wash beads twice with 200 µl 80% ethanol (freshly prepared).
- Remove all traces of ethanol with a pipette, then let tube stand for 5 mins (still on magnet) to allow ethanol to evaporate.
- Remove tube from magnet and resuspend beads in 17 µl RSB, then incubate 2 mins on bench.
- Replace tube on magnet and allow to clear. Transfer 15 µl to a fresh tube.

This DNA is now an Illumina ChIP-seq library.

2. QC library

Before sequencing, we recommend the following tests of library quantity & quality.

- Measure DNA quantity using the Qubit High Sensitivity kit, or an equivalent fluorometric method
- Control that adapter dimers have been removed, and that library is of expected size (adapters will add 122 bp to the original input) using the Bioanalyzer High Sensitivity reagents.
- After diluting library to 10 or 2 nM as recommended by Illumina for sequencing, control amplification of the generated library to a previous library of known performance by real-time PCR. See Illumina’s guidelines for details (Part # 11322363 Rev. C).
